# Diagnostic value and therapeutic efficacy of serum levels of pro-gastrin-releasing peptide precursor (ProGRP) and neuron-specific enolase (NSE) in patients with lung cancer

**DOI:** 10.5937/jomb0-50852

**Published:** 2025-08-21

**Authors:** Ping Wang, Mingjie Yu

**Affiliations:** 1 Bozhou Hospital of Traditional Chinese Medicine, Clinical Laboratory, Qiaocheng District, Bozhou, China; 2 The People's Hospital of Bozhou, Nuclear Medicine Department, Qiaocheng District, Bozhou, China

**Keywords:** small cell lung cancer, serum ProGRP, serum NSE, tumour marker, application value, rak malih ćelija pluća, serum ProGRP, serum NSE, tumor marker, primenjena vrednost

## Abstract

**Background:**

Primary lung cancer is one of the most prevalent malignant tumours in China. Small cell lung cancer (SCLC) is a highly malignant, undifferentiated tumour prone to metastasis and is usually diagnosed in its middle or late stages. Pro-gastrin-releasing peptide precursor (ProGRP) and neuron-specific enolase (NSE) tumour markers are recommended in the literature for early diagnosis. Objective: The purpose of this research is to probe the diagnostic value and therapeutic efficacy of serum levels of ProGRP and NSE in SCLC to enhance the level of clinical diagnosis.

**Methods:**

A total of 84 SCLC patients who were admitted to our hospital from December 2022 to March 2024 were included in the SCLC group. The NSCLC group consisted of 45 patients diagnosed with NSCLC, while the benign lung disease group consisted of 57 patients diagnosed with non-cancerous lung conditions. Furthermore, the healthy control group comprised 60 healthy individuals. The serum levels of ProGRP and NSE were compared across all four groups.

**Results:**

The SCLC group exhibited considerably elevated serum ProGRP and NSE levels compared to the healthy control group, benign lung disease group, and NSCLC group (P< 0.05). ProGRP and NSE values were higher in limited-stage SCLC than in extensive-stage SCLC (P < 0.05). The ROC curve displayed that the critical value of ProGRP for diagnosing SCLC was 136.49 pg/mL, the area under the curve (AUC) was 0.869, the sensitivity attained 80.00%, and the specificity reached 84.87%, indicating a better diagnostic efficacy than that of NSE (P< 0.05).

**Conclusions:**

The tumour markers ProGRP and NSE levels are of paramount significance for the clinical diagnosis and staging of SCLC patients. ProGRP is a more specific and sensitive tumour marker for SCLC than NSE and can be employed as an auxiliary diagnostic tool for SCLC. Thus, it is worth promoting ProGRP in a clinical setting.

## Abstract (sr)

**Uvod:**

Primarni rak pluća je jedan od najrasprostranjenijih zloćudnih tumora u Kini. Rak malih ćelija pluća (SCLC) je visoko maligni, nediferencirani tumor sklon metastazama i obično se dijagnostikuje u srednjim ili kasnim fazama. Tumor markeri peptid koji oslobađa progastrin (ProGRP) i neuron-specifična enolaza (NSE) su u literaturi preporučeni za rano dijagnostikovanje. Cilj: Svrha ovog istraživanja je da ispita dijagnostičku vrednost i terapeutski učinak nivoa ProGRP i NSE u serumu kod SCLC-a kako bi se poboljšao nivo kliničke dijagnoze.

**Metode:**

Ukupno 84 pacijenta sa SCLC-om koji su primljeni u našu bolnicu od decembra 2022. do marta 2024. su uključeni u grupu sa SCLC-om. Grupu sa NSCLC-om je činilo 45 pacijenata, dok je grupu sa benignim plućnim bolestima činilo 57 pacijenata sa benignim stanjima. Pored toga, grupa zdravih kontrolnih pacijenata se sastojala od 60 zdravih pojedinaca. Nivoi ProGRP-a i NSE-a u serumu su upoređivani među sve četiri grupe.

**Rezultati:**

Grupa sa SCLC-om je pokazala značajno povišene nivoe ProGRP-a i NSE-a u serumu u poređenju sa kontrolnom grupom, grupom sa benignim plućnim bolestima i grupom sa NSCLC-om (P< 0,05). Vrednosti ProGRP-a i NSE-a su bile više u ograničenom stadijumu SCLC-a nego u proširenom stadijumu SCLC-a (P< 0,05). ROC kriva je pokazala da je kritična vrednost ProGRP-a za dijagnostikovanje SCLC-a 136,49 pg/mL, da je površina ispod krive (AUC) 0,869, da je senzitivnost dostigla 80,00%, a specifičnost 84,87%, što ukazuje na bolju dijagnostičku efikasnost u poređenju sa NSE-om (P< 0,05).

**Zaključak:**

Tumor markeri ProGRP i nivo NSE-a su od najveće važnosti za kliničku dijagnozu i stadijum SCLC pacijenata. ProGRP je specifičniji i osetljiviji tumor marker za SCLC u poređenju sa NSE-om i može da se koristi kao pomoćni dijagnostički alat za SCLC. Stoga se preporučuje upotreba ProGRP-a u kliničkom okruženju.

## Introduction

ProGRP and NSE are recommended tumour markers for SCLC. Nevertheless, recent studies have reported that NSE has low sensitivity in the early stages of SCLC and may yield positive expressions in NSCLC and hemolysed samples, limiting its clinical utility. ProGRP is a newly discovered tumour marker, but its value in SCLC diagnosis still requires further validation. Therefore, our research intends to compare and analyse the application value of serum ProGRP and NSE in SCLC diagnosis, with the hope of offering some assistance in the early diagnosis and treatment of SCLC.

Primary lung cancer is among China's most frequently seen malignancies, which can be divided into two primary types: SCLC and NSCLC. SCLC is a type of neuroendocrine tumour that is highly malignant and undifferentiated and is characterised by its tendency to metastasise early in its development, accounting for 15%-20% of all lung cancer cases and commonly occurring in middle-aged and elderly populations [1] [2] [3]. During its initial stages, SCLC may not display any symptoms. However, in the middle and late stages of the disease, symptoms such as coughing, shortness of breath, weight loss, fatigue, pain, hemoptysis, and other related symptoms may present themselves. Severe cases may also present with symptoms such as Horner syndrome, carcinoid syndrome, or Cushing's syndrome [4] [5]. Under clinical circumstances, surgery is only considered for less than 5% of early-stage patients with tumours limited to pulmonary parenchyma. At the same time, limited-stage SCLC is primarily treated with concurrent chemoradiotherapy or sequential chemotherapy and radiotherapy, and extensive-stage SCLC is mainly treated with chemotherapy and then local or metastatic lesion therapy at a selected time point [6]. Reportedly, SCLC shows a poor prognosis due to its early rapid metastasis and fast growth, so early diagnosis and treatment are crucial for its prevention and management. Histopathological examination is the gold standard for diagnosing SCLC, but it is an invasive test for which patients have poor tolerance, making it unsuitable for clinical screening [7]. Therefore, seeking sensitive and accurate serum markers carries great clinical value in the early detection and diagnosis of SCLC and in improving patient treatment outcomes and survival.

Currently, the recommended tumour markers for SCLC primarily encompass ProGRP and NSE according to the guidelines [8]. NSE is an acid protease unique to neurons and neuroendocrine cells. High levels of NSE can be monitored in the serum of patients with tumours originating from neuroectodermal or neuroendocrine tissues [9]. Studies have disclosed that NSE is abnormally expressed in SCLC patients and is dramatically elevated in NSCLC patients. Among different tissue types of lung cancer tissues and serum, SCLC boasts the highest content of NSE [10]. However, recent reports have suggested that NSE demonstrates low sensitivity in the early detection of SCLC, and positive expressions quickly occur in NSCLC and hemolytic specimens, which limits its clinical application [11]. GRP is a gastrointestinal hormone in the average human brain, gastrointestinal neural fibres, and fetal lung neuroendocrine tissues. ProGRF) the precursor structure of GRF) is extensively present in non-gastric antrum tissues, nerve fibres, and neuroendocrine cells in the brain and lungs [12]. ProGRP is categorised into three molecular subtypes based on differences in partial amino acid residues, and they have a common C-terminal sequence of ProGRP Research experiments have provided evidence to support the claim that ProGRP can act as a new tumour marker for SCLC by indicating GRP's level and gene expression [13]. Nonetheless, its value in SCLC diagnosis still needs further confirmation.

Based on this, the present research retrospectively analysed the clinical data of SCLC patients, measured ProGRP and NSE levels in the patient serum, and evaluated their diagnostic values for SCLC patients. The aim is to offer more valuable insights into the treatment and prognosis of SCLC patients.

## Materials and methods

### General data

Staging criteria for lung cancer: According to the staging system of the Veterans Administration Lung Study Group (VALG) in the United States [14], SCLC is determined as a limited stage if the tumour tissues are present in the ipsilateral thoracic cavity, mediastinum, and supraclavicular areas, and as an extensive stage if the tumour tissues extend beyond the limited stage area.

### Inclusion and exclusion criteria

The inclusion criteria for SCLC and NSCLC groups were as follows: (1) Conforming to the diagnosis criteria indicated by the 2019 Clinical Diagnosis and Treatment Guidelines for Lung Cancer of the Chinese Medical Association [15]; (2) Eastern Cooperative Oncology Group (ECOG) score <2 points and expected survival time ≥3 months; (3) Age >18 years old; (4) At least one measurable lesion; (5) Pathological diagnosis of lung cancer confirmed by bronchoscopy or lung puncture biopsy.

Inclusion criteria for the benign lung disease group: Patients were diagnosed with benign lung diseases such as lung infection, pulmonary nodules, and pulmonary bullae based on clinical symptoms, physical signs, blood routine examinations, and chest X-ray examinations.

Exclusion criteria for all study subjects: (1) Patients with other malignant tumours; (2) Patients with severe gastrointestinal, liver, kidney, or cardiovascular diseases; (3) Patients with severe coagulation disorders or cardiovascular diseases; (4) Patients with mental disorders who were unable to cooperate with the clinical study.

### Specimen collection

The study harvested 3 mL of venous blood from the study subjects in a fasting state during the early morning. The collected samples were subjected to quiescence for 15-30 minutes, followed by centrifugation (3000 r/min, 15 min) at room temperature (RT). Subsequently, they were subjected to serum analysis. Samples that could not be promptly analysed were stored at -20°C until testing, with repeated freeze-thaw cycles and heating of the specimens strictly prohibited [16]. ProGRP and NSE determinations were performed on all specimens.

### Observation of Indicators

### Observation and recording of ProGRP and NSE concentrations in the serum of all groups

According to the manufacturer's instructions, the expression of ProGRP was analysed on the automated chemiluminescence immunoassay analyser (Mindray CL-6000i, China). The automatic electrochemiluminescence immune analyser Elec-sys2010 (Roche Diagnostics, Switzerland) was harnessed to examine NSE quantitatively. The critical value of NSE was set at 17 pg/mL.

### Observation and recording of ProGRP and NSE concentrations in limited- and extensive-stage SCLC

The recording method was the same as before.

### Evaluation of the diagnostic value

With the purpose of evaluating the diagnostic efficiency of serum ProGRP and NSE for small cell lung cancer (SCLC), the receiver operating characteristic (ROC) curve was employed. The ROC analysis compared the area under the curve (AUC), sensitivity, specificity, and Youden index.

### Evaluation of treatment methods and efficacy

Eighty-four SCLC patients received two cycles of chemotherapy consisting of etoposide and cisplatin. The specific administration method involved the following: Etoposide was administered at a dose of 0.1 g, added to 250 mL of 0.9% sodium chloride for intravenous drip in a light-avoiding manner, completed within 30 to 90 minutes from the 1st to the 5th day. Cisplatin (80 mg/mL) was diluted in 250-500 mL of 0.9% sodium chloride and intravenously transfused over 2-3 hours from the 1st to the 4th day. The cycle was repeated every 21 days for a total of 2 cycles.

According to the RECIST 1.1 criteria for solid tumours, established by the World Health Organization in 2009 [17], treatment efficacy was evaluated. Complete response (CR) indicates the absence of all target lesions, no new lesions, and normalisation of tumour markers for at least 4 weeks. Partial response (PR) is characterised by a reduction of at least 30% in the sum of the maximum diameters of the target lesions (for a minimum duration of 4 weeks). Stable disease (SD) refers to a reduction in the sum of the maximum diameters of the target lesions that do not meet the PR criteria or an increase that does not meet the PD criteria. Progressive disease (PD) is recognised as either the emergence of new lesions or an increase of 20% or more in the sum of the maximum diameters of the target lesions. The overall clinical response rate (RR) = (PR+CR) / total cases x 100%. The duration between the commencement of therapy and disease progression or patient death was defined as progression-free survival (PFS). The PFS of all study subjects was recorded to assess their prognosis [18].

### Assessment of the relationship between serum ProGRP and NSE levels and age, gender, smoking history, staging, etc., In SCLC patients

ProGRP and NSE levels In the serum of SCLC patients were quantitatively determined using the automated Chemiluminescence Immunoassay Analyzer (Mlndray CL-60001) and the automatic electrochemllumlnescence Immunoassay analyser Elecsys 2010 (Roche Diagnostics, Switzerland). The correlation between these levels and the patient's age, gender, smoking history, staging, distant metastasis, and tumour diameter was also evaluated.

### Statistical analysis

The data were analysed using the IBM SPSS21.0 software (SPSS Inc., Chicago, IL, USA). Measurement data were represented as (x±s). Moreover, *t*-tests were performed for comparisons between two groups, and a one-way analysis of variance (the SNK method) was taken for multiple-group comparison. Enumeration data were exhibited as (n, %). Univariate comparisons were made employing χ^2^. The ROC curve was capitalised to evaluate the ProGRP and NSE diagnostic value for SCLC. If *P*<0.05, variances held statistical significance.

## Results

Based on a retrospective analysis of clinical data from 84 SCLC patients and 45 NSCLC patients admitted to our hospital between December 2022 and March 2024, there were 55 male and 51 female patients In the SCLC group, Including 58 with limited-stage SCLC and 46 with extensive-stage SCLC. The NSCLC group Included 27 male and 18 female patients. In the benign lung disease group, there were 57 patients (34 males and 25 females) treated In our outpatient or Inpatient departments during the same period, Including those with pulmonary tuberculosis (15 cases), tuberculous pleurisy (8 cases), pulmonary Infection (14 cases), pulmonary cysts (12 cases), and chronic obstructive pulmonary disease (8 cases). The control group consisted of 60 healthy Individuals who underwent health checkups at our hospital and were randomly selected (35 males and 25 females). All patients understood the details of this study and voluntarily provided Informed consent by signing the document. There was no statistically significant difference In the general Information among the groups (*P*>0.05), Indicating comparability (see Table 1).

**Table 1:** Comparison of general data among the groups (n, %) (x±s).

General data		SCLC group<br>(n=84)	NSCLC group<br>(n=45)	Benign lung<br>disease group<br>(n = 57)	Healthy control<br>group<br>(n = 60)	*F/χ^2^*	*P* value
Gender (case)	Male	53	27	34	35	6.782	0.079
	Female	31	18	23	25		
Age (years old)	-	62.54±11.83	63.89±13.95	62.52±19.78	60.53±22.98	2.580	0.054
BMI (kg/m^2^)	-	24.23±6.88	25.98±6.76	24.24±7.43	24.22±7.38	2.580	0.055
Diameter of<br>tumour (cm)	≥5	49	32	-	-	2.048	0.152
	<5	35	13	-	-		
Smoking history<br>(case)	Yes	55	26	28	29	5.646	0.150
	No	29	19	29	31		
Family history<br>of tumour (case)	Yes	38	26	21	25	4.770	0.189
	No	46	19	36	35		

### Detection outcomes of serum ProGRP and NSE in each study group

Through observation of serum ProGRP and NSE concentrations In each group, we discovered that the serum levels of ProGRP (314.43±5.54 pg/mL) and NSE (124.54±3.84 pg/mL) In the SCLC group were greater than those In the healthy control group, the benign lung disease group, and the NSCLC group, with the difference being statistically significant (*P*<0.05). This finding denoted that statistical differences existed (*P*<0.05) In serum ProGRP and NSE levels among the four groups: healthy control, benign lung disease, SCLC, and NSCLC. ProGRP and NSE levels demonstrated a significant function In diagnosing SCLC patients (Table 2).

**Table 2:** Comparison of ProGRP and NSE concentrations in each group (x̄±s). Note: By contrast with the healthy control group, ①*P*<0.05 versus the benign lung disease group, ②*P*<0.05; vis-à-vis the NSCLC group, ③*P*<0.05.

Group	ProGRP (pg/mL)	NSE (pg/mL)
Healthy control (n = 60)	5.56±0.68	7.64±1.13
Benign lung disease (n = 57)	24.32±4.36^① ^	27.56±5.26^① ^
NSCLC (n=45)	56.68±6.32^①② ^	48.62±5.21^①② ^
SCLC (n=84)	214.43±61.54^①②③ ^	124.54±33.84^①②③ ^
*F*	461.859	433.761
*P *value	<0.001	<0.001

### Comparison of serum ProGRP and NSE levels in SCLC patients with different stages

Observation of ProGRP and NSE levels in the serum of SCLC patients at different stages suggested that the serum levels of ProGRP (268.35±4.66 pg/mL) and NSE (98.41±3.76 pg/mL) in extensive-stage SCLC were higher than those in limited-stage SCLC [ProGRP (154.42±4.55 pg/mL) and NSE (57.69±6.33 pg/mL)], with the difference deemed statistically significant (*P*<0.05).

### Diagnostic value of serum ProGRP and NSE levels in SCLC

**Figure 1:**
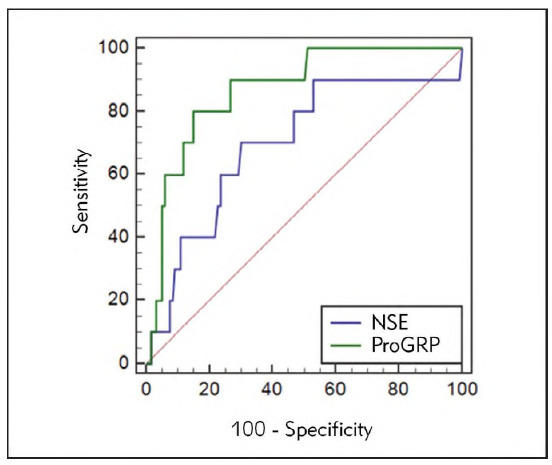
Diagnostic value of serum ProGRP and NSE levels in SCLC.

The diagnostic value of serum ProGRP and NSE levels in SCLC was evaluated by plotting ROC curves. The results indicated that the critical value of ProGRP for diagnosing SCLC was 136.49 pg/mL, with an AUC of 0.869, a sensitivity of 80.00%, and a specificity of 84.87% (*P*<0.05). The critical value of NSE for SCLC diagnosis was 76.87 pg/mL, with an AUC of 0.696, a sensitivity of 70.00%, and a specificity of 69.75% (P<0.05). Serum ProGRP demonstrated significantly higher values for AUC, sensitivity, and specificity in diagnosing SCLC than serum NSE, indicating that ProGRP is a better diagnostic marker for SCLC than NSE. See Table 3 Table 4 and Figure 1. 

**Table 3:** Comparison of ProGRP and NSE levels between limited-stage and extensive-stage SCLC (x̄±s).

Group	ProGRP (pg/mL)	NSE (pg/mL)
Limited-stage<br>SCLC (n = 38)	154.42±4.55	57.69±6.33
Extensive-stage<br>SCLC (n=46)	261.35±4.66	98.41 ±3.76
*T*	105.795	36.543
*P *value	<0.001	<0.001

**Table 4:** Diagnostic value of serum ProGRP and NSE levels in SCLC.

Indicator	Cut-off	AUC	Sensitivity	Specificity	Youden index	95%*CI*	*P*
ProGRP	>136.49 pg/mL	0.869	80.00%	84.87%	0.649	0.767-0.971	<0.001
NSE	>76.87 pg/mL	0.696	70.00%	69.75%	0.398	0.507-0.885	0.043

### The correlation between chemotherapy efficacy and serum ProGRP and NSE levels in SCLC patients

After receiving two cycles of chemotherapy and using the ROC threshold value as the grouping criterion, 45 SCLC patients were classified into the high ProGRP level group, and 59 were classified into the low ProGRP level group. Our data showed that clinical chemotherapy efficacy was better in the low ProGRP level group than in the high ProGRP level group (*P*<0.05). However, no statistically significant difference was found in progression-free survival (PFS) between the two groups (*P*> 0.05). Additionally, 40 SCLC patients were in the high NSE level group, while 41 were in the low NSE level group. There was no statistically significant difference in chemotherapy efficacy or PFS between these two groups (*P* >0.05), see Table 5. These findings indicate that SCLC patients with higher serum ProGRP levels had worse chemotherapy efficacy, suggesting that serum ProGRP levels could reflect chemotherapy efficacy in SCLC patients.

**Table 5:** The relationship between chemotherapy efficacy and serum ProGRP and NSE levels in SCLC patients (n, %; x±s). Complete response, CR; Stable disease, (SD); Partial Response, (PR); response rate, (RR); Progressive disease, (PD).

Group	Case	CR	PR	SD	PD	RR	*PFS*<br>(month, x̄±s)
High ProGRP level<br>group (> 136.49<br>pg/mL)	45	15 (33.33)	7 (15.56)	13 (28.89)	10 (22.22)	28 (71.79)	8.23±2.45
Low ProGRP level<br>group (< 136.49<br>pg/mL)	39	10 (25.64)	18 (46.15)	6 (15.38)	5 (12.82)	28 (71.79)	9.21 ±2.65
*T/χ^2^* value		9.707				4.550	1.760
*P* value		0.021				0.033	0.082
NSEHigh NSE level<br>group (>76.87<br>pg/mL)	40	12 (30.00)	18 (45.00)	6 (15.00)	4 (10.00)	30 (75.00)	6.57±2.58
NSELow NSE level<br>group (<76.87<br>pg/mL)	44	10 (22.73)	15 (34.10)	9 (20.45)	10 (22.73)	25 (56.82)	6.73±5.24
*T/χ^2^* value		3.443				3.064	0.253
*P* value		0.528				0.080	0.801

### The association between SCLC patients' serum ProGRP and NSE levels and their age, gender, smoking history, and staging

Further analysis was conducted to investigate the correlation between the serum levels of ProGRP and NSE in SCLC patients and their age, gender, smoking history, and staging. The outcomes hinted that the levels of ProGRP and NSE had no evident correlation with age, gender, or smoking status (*P*>0.05) but were obviously related to staging. Furthermore, the levels of ProGRP and NSE in extensive-stage SCLC [ProGRP (227.72±45.41) pg/mL; NSE (136.57±32.87) pg/mL] were notably higher than those in limited-stage SCLC [ProGRP (198.34±64.41) pg/mL; NSE (109.98±31.33) pg/mL] (*P*<0.05). ProGRP and NSE levels were evidently higher in SCLC patients with lymph node metastasis and tumour diameter ≥5 cm than in those without lymph node metastasis and tumour diameter <5 cm. The observed differences contained statistical significance (*P*<0.05). The study results are presented in Table 6.

**Table 6:** The association between SCLC patients' serum ProGRP and NSE levels and their age, gender, smoking history, staging, etc (x̄±s).

General<br>information		Case	ProGRP (pg/mL)	*T*	*P* value	NSE (pg/mL)	T	*P* value
Gender<br>(case)	Male	53	215.59±62.87	0.220	0.826	123.32±32.36	0.443	0.659
	Female	31	212.45±63.33			126.63±34.24		
Age	<60	33	212.90±62.12	0.184	0.855	122.54±34.12	0.448	0.655
	>60	51	215.42±61.03			125.83±32.06		
Smoking status	Yes	55	214.65±64.88	0.043	0.966	123.53±32.24	0.392	0.696
	No	29	214.01 ±65.43			126.46±33.27		
Staging	Limited-stage<br>SCLC	38	198.34±64.41	2.445	0.017	109.98±31.33	3.769	<0.001
	Extensive-stage<br>SCLC	46	227.72±45.41			136.57±32.87		
Distant metastasis	No	39	199.89±62.46	2.398	0.019	115.56±32.98	2.386	0.019
	Yes	45	227.03±40.23			132.32±31.34		
Diameter of<br>mour	<5 cm	49	202.78±62.87	2.285	0.025	114.67±34.52	3.429	0.001
	≥5 cm	35	230.74±42.34			138.56±25.85		

## Discussion

SCLC is a type of neuroendocrine tumour marked by a high degree of malignancy and rapid disease progression, with early symptoms often being inconspicuous, and it can manifest the characteristics of neuroendocrine cells [19]. As SCLC patients do not show significant symptoms in the initial phase of the illness, the majority of them receive a diagnosis at an advanced stage, posing a challenge to cure. Therefore, rapid screening and diagnosis of SCLC are of great medical and clinical value for healthcare professionals.

As reported, both NSE and ProGRP can be applied in SCLC diagnosis, but their diagnostic efficiency differs [20]. NSE is widely believed to be the preferred marker for various neural and neuroendocrine tissue cancers, as it is present in neural and neuroendocrine cells. Nevertheless, its clinical application is restricted due to its high false positive rate in the early stages of SCLC and the potential for positive outcomes in non-SCLC and hemolytic specimens [21] [22] [23]. The latest studies have unveiled that GRP may act as an autocrine growth factor for a subgroup of SCLC tumour cells, enabling rapid tumour growth. ProGRF) a proliferation factor of SCLC, is a product encoded by genes with stable plasma levels [24] [25]. Experimental studies have demonstrated that ProGRP levels represent GRP levels and GRP gene expression, making it a novel tumour marker for SCLC. ProGRP has been increasingly applied and promoted in China in the past two years [26].

In the present research, we measured the levels of ProGRP and NSE in the serum of 84 SCLC patients, 45 NSCLC patients, 57 patients with benign lung diseases, and 60 healthy individuals. ProGRP and NSE levels in the serum of SCLC patients were higher than those of the healthy control group, the benign lung disease group, and the NSCLC group. Furthermore, the levels of ProGRP and NSE were elevated in patients with limited-stage SCLC versus those with extensive-stage SCLC, and the differences between groups had statistical significance. These findings reflected that ProGRP and NSE, which served as tumour markers, exhibited high profiles in SCLC patients and were inextricably associated with the severity of the disease. Thus, ProGRP and NSE could be utilised as critical diagnostic indicators for SCLC in clinical practice, which is aligned with the conclusion of Winther B et al. [27]. This can be attributed to NSE generally being present in the tumour cells of neuroendocrine tissues. When the tumour cells containing NSE rupture, NSE spreads into the blood, contributing to higher levels of NSE in the serum of SCLC patients than in other patients or healthy individuals [28]. Additionally, SCLC cells can generate and secrete GRP per se to modulate their own growth in an autocrine mode and release GRP into the tissues to stimulate the proliferation and unlimited growth of the tumour by binding to GRP receptors on the cell membrane, giving rise to an increase in the serum levels of ProGRP [29].

To further establish the value of serum ProGRP and NSE in assessing disease status in SCLC patients, this research plotted ROC curves for SCLC diagnosis employing serum ProGRP and NSE. As demonstrated by the outcomes, the sensitivity, specificity, and Youden index of ProGRP in diagnosing SCLC were evidently higher than those of NSE. These findings reflected that serum ProGRP and NSE exhibited a high diagnostic efficacy for assessing SCLC patients' illness state and could serve as effective markers for predicting disease progression. Moreover, ProGRP appeared to be more effective than NSE in terms of diagnostic efficacy.

In clinical practice, chemotherapy Is the mainstay of treatment for patients with SCLC, and multiple cycles of chemotherapy are required [30] [31]. Using the ROC threshold as the grouping criterion, the clinical therapeutic effect of SCLC patients with different serum ProGRP and NSE levels was compared. It was disclosed that after two cycles of chemotherapy, the clinical chemotherapy efficacy was higher In the low-level ProGRP group than In the high-level ProGRP group, while the low-level NSE group exhibited a higher PFS level than the high-level NSE group (*P*<0.05). These findings hinted that SCLC patients with higher serum ProGRP levels showed poorer chemotherapy efficacy and that serum ProGRP levels could reflect the chemotherapy efficacy of SCLC patients.

A meta-analysis of 27 studies found that serum ProGRP Is a reliable blomarker for diagnosing small cell lung cancer, with a pooled sensitivity of 75.4% and specificity of 94.5%, Indicating a strong ability to detect the disease. The results, which Included a diagnostic odds ratio of 53.1 and an area under the curve of 0.91, suggest that proGRP can be widely used In clinical settings to Identify lung cancer patients [32]. Based on the study of Roselk et al. [33] In 2023, 290 cases of lung neuroendocrine neoplasms (LNENs) found that ProGRP Is an effective blomarker for diagnosis, with median levels significantly higher In LNEN patients (136.4 pg/mL) compared to controls (6.5 pg/mL). Our study also confirmed these results. However, this might not always stay as specific as mentioned In the literature. A case report of an 86-year-old woman presented with symptoms and Imaging findings suggestive of small-cell lung cancer showed a significantly elevated ProGRP level of 888 pg/mL but was ultimately diagnosed with an atypical carcinoid tumour [34].

A study by Sun et al. [35] on 82 patients with lung cancer found that serum levels of NSE decreased significantly after radiotherapy, suggesting that these blomarkers can be used to evaluate the effectiveness of treatment. The study also found that NSE levels were lower In patients who did not experience recurrence or metastasis (n = 54) compared to those who did (n=28), Indicating that these blomarkers may have predictive value for prognosis [35]. While we have similarly compared these disease progression or treatment statistics, we did not find such statistically significant findings.

Moreover, to dig deeper Into the relationship between the tumour markers ProGRP and NSE and clinical pathological features, we gathered basic Information such as age, gender, smoking history, and tumour stage of SCLC patients and examined the variations In serum ProGRP and NSE levels In SCLC patients with distinct characteristics. As a result, no substantial statistical difference was discovered In serum ProGRP and NSE levels between SCLC patients with different genders, ages, and smoking histories. Nevertheless, ProGRP and NSE serum levels were dramatically elevated In patients with extensive-stage SCLC, who had lymph node metastasis and a tumour diameter of at least 5 cm, vis-à-vis patients with limited-stage SCLC who had no lymph node metastasis and a tumour diameter less than 5 cm. Tumour diameter reflects the current growth status of the tumour, while clinical staging and distant metastasis tell the location of the primary lesion and the extent of metastasis. These findings disclosed that ProGRP and NSE might have significant reference value In SCLC's occurrence, development, and metastasis.

To conclude, the levels of tumour markers ProGRP and NSE carry Important clinical significance for diagnosing and staging SCLC patients. ProGRP Is a more specific and sensitive tumour marker for SCLC In contrast to NSE and can be adopted as an adjunct diagnostic tool for SCLC, which Is worth promoting In a clinical setting. However, this study presented here also has certain limitations, such as the Inclusion time and study population, which did not allow for a more In-depth exploration of the Impact of ProGRP and NSE combined on diagnosing SCLC patients. This will be further addressed In future research.

## Conclusion

To summarise, the expression levels of tumour markers ProGRP and NSE In the serum of SCLC patients are higher than those In NSCLC patients, patients with benign lung diseases, and healthy Individuals. Furthermore, ProGRP and NSE serum levels Increase with tumour stage progression. Additionally, ProGRP and NSE serum levels vary among SCLC patients with different stages, lymph node metastasis, and tumour diameters. Patients with advanced stages of SCLC, lymph node metastasis, and larger tumour sizes exhibit elevated levels of serum tumour markers. Moreover, compared to NSE, ProGRP Is a more specific and sensitive tumour marker for SCLC. This research provides new Insights for the diagnosis and prognosis evaluation of SCLC patients and warrants further clinical promotion and reference.

## Dodatak

### Conflict of interest statement

All the authors declare that they have no conflict of Interest In this work.

## References

[1] van Meerbeeck J P, Fennell D A, de Ruysscher D K M (2011;Nov 12). Small-cell lung cancer. Lancet.

[2] Wang Y, Zou S, Zhao Z, Liu P, Ke C, Xu S (2020;Aug). New insights into small-cell lung cancer development and therapy. Cell Biol Int.

[3] Herbst R S, Morgensztern D, Boshoff C (2018;Jan 24). The biology and management of non-small cell lung cancer. Nature.

[4] Zugazagoitia J, Paz-Ares L (2022;Feb 20). Extensive-Stage Small-Cell Lung Cancer: First-Line and Second-Line Treatment Options. J Clin Oncol.

[5] Ganti A K P, Loo B W, Bassetti M, Blakely C, Chiang A, D'amico T A, et al (2021;Dec). Small Cell Lung Cancer, Version 2.2022, NCCN Clinical Practice Guidelines in Oncology. J Natl Compr Canc Netw.

[6] Bernhardt E B, Jalal S I (2016). Small Cell Lung Cancer. Cancer Treat Res.

[7] Gazdar A F, Bunn P A, Minna J D (2017;Dec). Small-cell lung cancer: What we know, what we need to know and the path forward. Nat Rev Cancer.

[8] Kiseli M, Caglar G S, Yarci G A, Tasci T, Candar T, Akincioglu E, Pabuccu E G, Boran N, Tulunay G, Umudum H (2018). Pro-Gastrin Releasing Peptide: A New Serum Marker for Endometrioid Adenocarcinoma. Gynecol Obstet Invest.

[9] Fang L, Huang Z, Lin Y, Fu J, Liang X, Liu F (2018;Jul 1). Clinical Application of Pro-Gastrin-Releasing Peptide. Clin Lab.

[10] Ueland T, Gullestad L, Kou L, Aukrust P, Anand I S, Broughton M N, Mcmurray J J, van Veldhuisen D J, Warren D J, Bolstad N (2018;Dec). Pro-gastrin-releasing peptide and outcome in patients with heart failure and anaemia: Results from the RED-HF study. ESC Heart Fail.

[11] Isgrô M A, Bottoni P, Scatena R (2015). Neuron-Specific Enolase as a Biomarker: Biochemical and Clinical Aspects. Adv Exp Med Biol.

[12] Haque A, Ray S K, Cox A, Banik N L (2016;Jun). Neuron specific enolase: A promising therapeutic target in acute spinal cord injury. Metab Brain Dis.

[13] Xu C, Luo Y, Li S, Li Z, Jiang L, Zhang G, Owusu L, Chen H (2019;Nov 29). Multifunctional neuron-specific enolase: Its role in lung diseases. Biosci Rep.

[14] Chen K N (2016;Jun 20). Small Cell Lung Cancer and TNM Staging. Zhongguo Fei Ai Za Zhi.

[15] Avasarala S K, Rickman O B (2022;Jun). Endobronchial Therapies for Diagnosis, Staging, and Treatment of Lung Cancer. Surg Clin North Am.

[16] Dagur P K, Mccoy J (2015;Jul). Collection, Storage, and Preparation of Human Blood Cells. Curr Protoc Cytom.

[17] Eisenhauer E A, Therasse P, Bogaerts J, Schwartz L H, Sargent D, Ford R, Dancey J, Arbuck S, Gwyther S, Mooney M, Rubinstein L, Shankar L, Dodd L, Kaplan R, Lacombe D, Verweij J (2009;Jan 1). New response evaluation criteria in solid tumours: Revised RECIST guideline (version 1.1). Eur J Cancer.

[18] Tran S, Bielle F (2022;Nov 1). WHO 2021 and beyond: New types, molecular markers and tools for brain tumor classification. Curr Opin Oncol.

[19] Wang W Z, Shulman A, Amann J M, Carbone D P, Tsichlis P N (2022;Nov). Small cell lung cancer: Subtypes and therapeutic implications. Semin Cancer Biol.

[20] Takada M, Kusunoki Y, Masuda N, Matui K, Yana T, Ushijima S, Iida K, Tamura K, Komiya T, Kawase I, Kikui N, Morino H, Fukuoka M (1996;May). Pro-gastrin-releasing peptide(31-98) as a tumour marker of small-cell lung cancer: Comparative evaluation with neuron-specific enolase. Br J Cancer.

[21] Ischia J, Patel O, Sethi K, Nordlund M S, Bolton D, Shulkes A, Baldwin G S (2015;May). Identification of binding sites for C-terminal pro-gastrin-releasing peptide (GRP)-derived peptides in renal cell carcinoma: A potential target for future therapy. BJU Int.

[22] Echeverría-Palacio C M, Agut T, Arnaez J, Valls A, Reyne M, Garcia-Alix A (2019;Dec). Neuron-Specific Enolase in Cerebrospinal Fluid Predicts Brain Injury After Sudden Unexpected Postnatal Collapse. Pediatr Neurol.

[23] Topuzova M P, Alekseeva T M, Panina E B, Vavilova T V, Kovzelev P D, Portik O A, Skoromets A A (2019). The possibility of using neuron-specific enolase as a biomarker in the acute period of stroke (Russian). Zh Nevrol Psikhiatr Im S S Korsakova.

[24] Sosnin D, Gal K K R, Khovaeva Y B, Gil M A (2022;Jul). Comparative Content of Neuron-Specific Enolase in Human Blood Serum and Seminal Plasma. Bull Exp Biol Med.

[25] Kamata K, Uchida M, Takeuchi Y, Takahashi E, Sato N, Miyake Y, Okubo M, Kodama T, Yamaguchi K (1996;Jul). Increased serum concentrations of pro-gastrin-releasing peptide in patients with renal dysfunction. Nephrol Dial Transplant.

[26] Dong J, Tong S, Shi X, Wang C, Xiao X, Ji W, Sun Y (2021;Jan 5). Progastrin-Releasing Peptide Precursor and Neuron-Specific Enolase Predict the Efficacy of First-Line Treatment with Epidermal Growth Factor Receptor (EGFR) Tyrosine Kinase Inhibitors Among Non-Small-Cell Lung Cancer Patients Harboring EGFR Mutations. Cancer Manag Res.

[27] Wojcik E, Kulpa J K, Sas-Korczynska B, Korzeniowski S, Jakubowicz J (2008;Sep-Oct). ProGRP and NSE in therapy monitoring in patients with small cell lung cancer. Anticancer Res.

[28] Lawi Z K, Alkhammas A H, Elerouri M, Ben A I, Al-Shuhaib M B S (2023). Two co-inherited SNPs of the telomerase reverse transcriptase (TERT) gene are associated with Iraqi patients with lung cancer. J Med Biochem.

[29] Rossetti C, Abdel Q A, Halvorsen T G, Sellergren B, Reubsaet L (2014;Nov 26). Antibody-Free Biomarker Determination: Exploring Molecularly Imprinted Polymers for Pro-Gastrin Releasing Peptide. Anal Chem.

[30] Marangos P J, Schmechel D E (1987). Neuron Specific Enolase, A Clinically Useful Marker for Neurons and Neuroendocrine Cells. Annu Rev Neurosci.

[31] Endo T, Hasegawa T, Tezuka Y, Kanai Y, Otani S, Yamamoto S, Tetsuka K, Sato Y, Endo S, Sohara Y (2008;Oct). Atypical pulmonary carcinoid tumor with abnormal elevation of serum gastrin-releasing peptide precursor: Report of a case. Kyobu Geka.

[32] Lv P, Wang Y, Huang L, Wang F, Zhou G, Ma H (2017). Meta-analysis of serum gastrin-releasing peptide precursor as a biomarker for diagnosis of small cell lung cancer. Asian Pacific Journal of Cancer Prevention: APJCP.

[33] Rosiek V, Kogut A, Kos-Kudła B (2023;Jun 22). Pro-Gastrin-Releasing Peptide as a Biomarker in Lung Neuroendocrine Neoplasm. Cancers (Basel).

[34] Nakagawa N, Kawakami M, Suzuki M, Noguchi S, Mitani A, Tanaka G, Shinozaki-Ushiku A, Nagase T (2023;Jan 1). Pulmonary carcinoid tumour with remarkably high levels of pro-gastrin-releasing peptide: A case report. Respir Med Case Rep.

[35] Sun L, Shao Q (2023;May 19). Expression changes and clinical significance of serum neuron-specific enolase and squamous cell carcinoma antigen in lung cancer patients after radiotherapy. Clinics (Sao Paulo).

